# Identification of Inflammatory and Disease-Associated Plasma Proteins that Associate with Intake of Added Sugar and Sugar-Sweetened Beverages and Their Role in Type 2 Diabetes Risk

**DOI:** 10.3390/nu12103129

**Published:** 2020-10-14

**Authors:** Stina Ramne, Isabel Drake, Ulrika Ericson, Jan Nilsson, Marju Orho-Melander, Gunnar Engström, Emily Sonestedt

**Affiliations:** Department of Clinical Sciences Malmö, Lund University, 214 28 Malmö, Sweden; isabel.drake@med.lu.se (I.D.); ulrika.ericson@med.lu.se (U.E.); jan.nilsson@med.lu.se (J.N.); marju.orho-melander@med.lu.se (M.O.-M.); gunnar.engstrom@med.lu.se (G.E.); emily.sonestedt@med.lu.se (E.S.)

**Keywords:** added sugar, sugar-sweetened beverages, inflammation, inflammatory proteins, proteomics, type 2 diabetes

## Abstract

It has been suggested that high intake of added sugar and sugar-sweetened beverages (SSBs) increase the level of circulating inflammatory proteins and that chronic inflammation plays a role in type 2 diabetes (T2D) development. We aim to examine how added sugar and SSB intake associate with 136 measured plasma proteins and C-reactive protein (CRP) in the Malmö Diet and Cancer–Cardiovascular Cohort (*n* = 4382), and examine if the identified added sugar- and SSB-associated proteins associate with T2D incidence. A two-step iterative resampling approach was used to internally replicate proteins that associated with added sugar and SSB intake. Nine proteins were identified to associate with added sugar intake, of which only two associated with T2D incidence (*p* < 0.00045). Seven proteins were identified to associate with SSB intake, of which six associated strongly with T2D incidence (*p* < 6.9 × 10^−8^). No significant associations were observed between added sugar and SSB intake and CRP concentrations. In summary, our elucidation of the relationship between plasma proteome and added sugar and SSB intake, in relation to future T2D risk, demonstrated that SSB intake, rather than the total intake of added sugar, was related to a T2D-pathological proteomic signature. However, external replication is needed to verify the findings.

## 1. Introduction

Different dietary components or food items could serve either as pro-inflammatory or anti-inflammatory, and the link between diet and chronic low-grade inflammation could be either direct or mediated through other factors, such as the gut microbiota [1]. The main direct pro-inflammatory effects of diet are induced by positive energy balance and accumulating adiposity [2]. Further, epigenetic changes or excess formulation of reactive oxygen species are examples of mechanisms in which the nutrients and foods we consume may promote inflammation [2].

High intake of added sugars and sugar-sweetened beverages (SSBs) has been hypothesized to promote chronic inflammation. Increased levels of inflammatory markers after high sugar interventions have been observed in a few randomized trials [3,4], while not in other trials [5,6,7]. The stress of a high postprandial plasma glucose is suggested as an additional potential reason [1]. Glycemic index and load have by some been observed to associate with inflammatory markers, but the overall evidence is inconclusive [8]. However, this is highly dependent on which inflammatory biomarkers are assessed [1]. Therefore, it is of great importance to investigate a wide range of inflammatory and disease-associated plasma proteins. Among the typical inflammatory markers, C-reactive protein (CRP) is best studied in relation to intake of added sugar and SSBs, and there is a lack of studies investigating other disease-associated plasma proteins.

SSB intake has in meta-analyses been shown to associate with increased type 2 diabetes (T2D) risk [9,10,11], while epidemiological investigations of sugar intake and T2D risk often show no association [12,13,14,15,16,17,18,19,20,21,22]. As far as we are aware, only three out of 14 studies have shown a significant positive association with T2D incidence [16,23,24]. These associations were, however, only found when intake of fructose or glucose were studied specifically, never when intake of sucrose, total sugar or added sugar were studied. Some studies have even shown significant inverse associations [23,25].

The role of chronic inflammation for T2D development is well acknowledged [26,27], and several meta-analyses have observed increased incidence in T2D with higher levels of circulating inflammatory markers [28,29].

All in all, a role of chronic inflammation as a link between added sugar and SSB intake and T2D risk is hypothesized, but associations between added sugar and SSB intake and both inflammatory proteins and T2D are inconsistent in the epidemiological research and limited to a few inflammatory proteins. Therefore, the aim of this explorative study was to examine how added sugar or SSB intake associate with inflammatory and disease-associated plasma proteins and CRP, and if the identified added sugar- and SSB-associated proteins prospectively associate with incidence of T2D.

## 2. Materials and Methods

### 2.1. Study Sample

The Malmö Diet and Cancer–Cardiovascular Cohort (MDC-CC) is a deeply phenotyped sub-cohort within the population-based prospective Malmö Diet and Cancer (MDC) cohort, conducted in the city of Malmö in southern Sweden between 1991 and 1996. Participants were recruited by advertisements in public areas and by a personal invitation letter sent to all men born between 1923 and 1945 and all women born between 1923 and 1950 inhabiting the Malmö city area. From the recruited participants in MDC, 6103 were randomly recruited to the MDC-CC during 1991–1994 and fasting blood samples were obtained from the 5540 participants. In total, 4865 blood samples were sent for analysis of plasma proteins, out of which 4742 analyzed samples passed quality control. From these, 301 individuals with a diagnosed diabetes or cardiovascular disease at baseline were excluded from the current study sample, as well as those with missing data on dietary intake (*n* = 26) or model covariates (*n* = 33), resulting in a total sample of 4382 participants. All participants signed written informed consent prior to entering the study, which was approved by the Lund University Ethical Committee (LU 51/90) in accordance with the Declaration of Helsinki.

### 2.2. Dietary Intake Assessment

To assess dietary intake, a combination of a seven-day food record of all cooked meals, cold beverages (including SSBs) and dietary supplements; a 168 item food frequency questionnaire covering all food items belonging to non-cooked meals spanning over the previous year; and a dietary interview elucidating food choices, cooking methods and portion sizes (with the help of a booklet of photographs of various portions sizes) were applied. The portion size estimation in the food frequency questionnaire was also aided by the booklet of photographs of various portion sizes. The energy and nutrient content of the collected food intake data were obtained from the MDC food database, which was based upon the Swedish national food database from the Swedish National Food Agency. Added sugar intake was estimated from the sum of sucrose and monosaccharides, subtracted for the sum of sucrose and monosaccharides naturally occurring in fruit, vegetables and fruit juices. This has been described in detail in a previous publication [30]. Added sugar intake was investigated as the percentage of non-alcohol energy intake (E%), both as a continuous variable and categorized into six previously studied categories [30]. The E% intake coming from SSBs was estimated on the assumption that all SSBs have a mean sugar content of 10 g per 100 g, based on that the sugar content in soft drinks varies between 10–13 g/100 g and cordial/squash contains approximately 8 g/100 g sugar. The contribution to the added sugar intake (E%) from SSB intake was also calculated. For statistical analyses, the SSB intake (E%) variable was either log transformed (with a constant of 0.1 added), due to its skewed distribution and large proportions of zero consumers, or divided into five categories of 0E%, >0–2E%, >2–3E%, >3–5E% and >5E%.

### 2.3. Plasma Protein Assessment

Fasting blood samples from baseline examinations were stored at −80 °C until analysis of plasma protein concentrations in 2015. A total of 149 plasma proteins were measured by the SciLifeLab (Uppsala, Sweden) with the Olink Proseek Multiplex proximity extension assays Cardiovascular 1 and Oncology 1 (Olink Proteomics, Uppsala, Sweden). For each protein, the proximity extension technology uses pairs of antibodies marked with oligonucleotides that bind pair-wise with the protein to be measured. When the oligonucleotides reach proximity, paired DNA strings are formed, which are quantified with qPCR. Values are presented as normalized protein expression units (real-time qPCR cycle values on the log2 scale). For statistical analysis, we excluded those proteins that were available in less than 75% of the individuals in the present study sample (*n* = 13), giving 136 plasma proteins analyzed at study samples ranging between 3351 and 4382 individuals. A full list of all 136 plasma proteins is available in Table S1.

Measurements of CRP were performed by the Tina-quant^®^ CRP latex high sensitivity assay (Roche Diagnostics, Basel, Switzerland) on an ADVIA 1650 Chemistry System (Bayer Healthcare, Tarrytown, NY, USA). The values are the means of measurements read in 6 s intervals over 1 min after 5 min of incubation. Within our study sample (*n* = 4382), measured CRP was available in 4291 individuals.

### 2.4. Covariate Assessment

In a self-administered questionnaire, medical history, medication use, socioeconomic factors such as education (assessed on a five-level scale) and lifestyle factors were assessed. The lifestyle factors included leisure time physical activity (LTPA) (five categories of metabolic equivalent hours per week based on the time spent engaging in 17 different activities and their metabolic equivalent intensity factor), alcohol habits (quintiles of consumers with one category of non-consumers) and smoking status (current smokers, former smokers and never smokers). A trained nurse measured body weight and height. Body mass index (BMI) was calculated as weight (kg)/height (m)^2^. Fasting plasma glucose was measured using standard procedures.

Low and high energy reporters were determined using Goldberg and Black’s cutoffs for the discrepancy between reported energy intake and estimated energy requirements [31]. Additionally, past diet changers were identified at baseline with the questionnaire question “Have you substantially changed your eating habits because of illness or some other reasons?”.

### 2.5. Outcome Assessment

Participants were followed from baseline examinations until incidence of T2D, date of either death or emigration or until 31 December 2016, whichever came first. Diabetes diagnosis was ascertained by the Swedish National Diabetes Register, the regional Diabetes 2000 register of the Scania region, the Malmö HbA1c (glycated hemoglobin) register, the Swedish in- and outpatient registers, the Swedish cause of death register and the Swedish drug prescription register, or in the re-examinations of the MDC-CC in 2007–2012 and the Malmö Preventive Project in 2002–2006 as previously described [32]. Dates of physician-made diagnosis were obtained from the Swedish National Diabetes Register and the Diabetes 2000 register. The Malmö HbA1c register identified individuals with two or more HbA1c measurements ≥ 6.0%. The in- and outpatient registers and the cause of death register identified those with the ICD10 codes E10–E14 and O244–O249, and the drug prescription register identified those who have obtained anti-diabetic medication. In our study sample, a total of 767 individuals were considered as incident diabetes cases, out of which 17 were confirmed to not be of type 2 and 310 were confirmed to be of type 2. For the remaining 440 diabetes cases, type was not specified. However, due to the high age of the population, these diabetes cases were assumed to be of type 2, resulting in a total of 750 cases of T2D.

### 2.6. Statistical Analyses

Statistical analyses were performed in Stata/SE (version 15, StataCorp LLC, College Station, TX, USA). Baseline characteristics were examined across six categories of added sugar intake and across five categories of SSB intake. Categorical variables were expressed as percentages and continuous variables were expressed as mean (SD) or median (IQR).

Associations between added sugar and SSB intake (E%, continuous variables, SSB log transformed) and plasma proteins were studied with multivariable linear regression, adjusted for age, sex, season, screening date, total energy intake, education level, smoking status, alcohol habits and LTPA. For internally replicating our discovery of proteins associating with added sugar and SSB intake, we adopted a two-step iterative resampling (TSIR) approach based on reasoning by Kang et al. [33]. The authors concluded that for gene discovery in genome-wide association studies, a 70:30 split into discovery and replication cohort and a replication in 20 out of 100 iterations provide satisfying balance between risk of type 1 and type 2 errors. In our study, the study sample was divided into 100 random discovery cohorts (2/3 of the study sample) and the remaining (1/3) was used as replication cohorts. The first step was performed in the discovery cohorts, where we identified the proteins that associated with added sugar and SSB intake (E%) at three different significance levels: α_1_ < 0.05, α_1_ < 0.01 and α_1_ corresponding to *p*-values adjusted for false discovery rate (FDR) of 0.05 using the Benjamini–Hochberg method. In step two, the identified proteins’ associations with added sugar and SSBs were then examined in the replication cohorts, where an α_2_ of 0.05 was always applied as cutoff. A protein was considered internally replicated if it was associated in the same direction in both the discovery and replication cohort in at least 20 out of 100 of the randomly generated discovery and replication cohorts. For plotting purposes, we standardized the added sugar and the logSSB variables to make ß-coefficients comparable.

Longitudinal associations between internally replicated plasma proteins with T2D incidence were examined using Cox proportional hazards regression with standardized proteins and using follow-up time as the time metric. The model was adjusted for age, sex, education level, smoking status, alcohol habits and LTPA, as well as in additional models with further adjustment for baseline BMI, fasting glucose and CRP. A Bonferroni corrected *p*-value of 0.003 (0.05/16) was applied as the significance level in this analysis of 16 proteins. We also studied potential interactions between the 136 plasma proteins (standardized) and added sugar and logSSB intake on T2D risk, in which the following diet-related covariates were further included in the regression models: total energy intake, season and start date. In these interaction analyses, Bonferroni corrected *p*-values of 0.05/136 = 0.00037 were applied. Correlations between all TSIR-identified plasma proteins were studied with pair-wise correlations, and pathways between the identified proteins were explored using the STRING database 11.0 [34].

The association between categories of added sugar and SSB intake and CRP (linear regression) and T2D incidence (Cox proportional hazards regression) were adjusted for age, sex, season, screening date, total energy intake, education level, smoking status, alcohol habits and LTPA. The CRP variable was log transformed due to the skewed nature of it. The predicted marginal means of CRP levels across intake categories were exponentiated back for presentation. We further analyzed the interaction effect of CRP on added sugar and SSB intake (in categories) on their association with T2D incidence. Plotting of interaction effects were performed using marginal predictions at five levels of CRP; −2SD, −1SD, mean, +1SD and +2SD and with mean-centered continuous covariates. Sensitivity analyses excluding low and high energy reporters and past diet changers were performed on the associations between added sugar and SSB intake and T2D and CRP.

## 3. Results

### 3.1. Baseline Characteristics

The study population of 4382 participants had a mean age of 57 years (range 45–68) and mean BMI of 25.5 kg/m^2^. Added sugar intake contributed on average to 10% of the energy intake, out of which 10% on average came from intake of SSBs. However, 46% of the study population had not reported any intake of SSBs in the seven-day food record, making the median contribution of SSBs to the added sugar intake <1%. As presented in Table 1, with increasing added sugar intake, the proportion of individuals with a university degree and high alcohol consumers appeared to be decreasing. The proportions of current smokers and those eventually getting a T2D diagnosis, as well as fasting glucose and CRP levels, were the highest in both the highest and lowest intake groups of added sugar. BMI appeared to be decreasing with increasing added sugar intake, while reported total energy intake and intake of SSBs were increasing. Table 2 shows how the proportion of women, individuals with a university degree and high alcohol consumers appears to decrease with increasing SSB intake. BMI, fasting glucose, CRP and T2D incidence appear to increase with increasing SSB intake, as well as total energy intake, added sugar intake and contribution of SSBs to added sugar intake.

### 3.2. Plasma Proteins Associated with Added Sugar Intake

**Figure 1:**
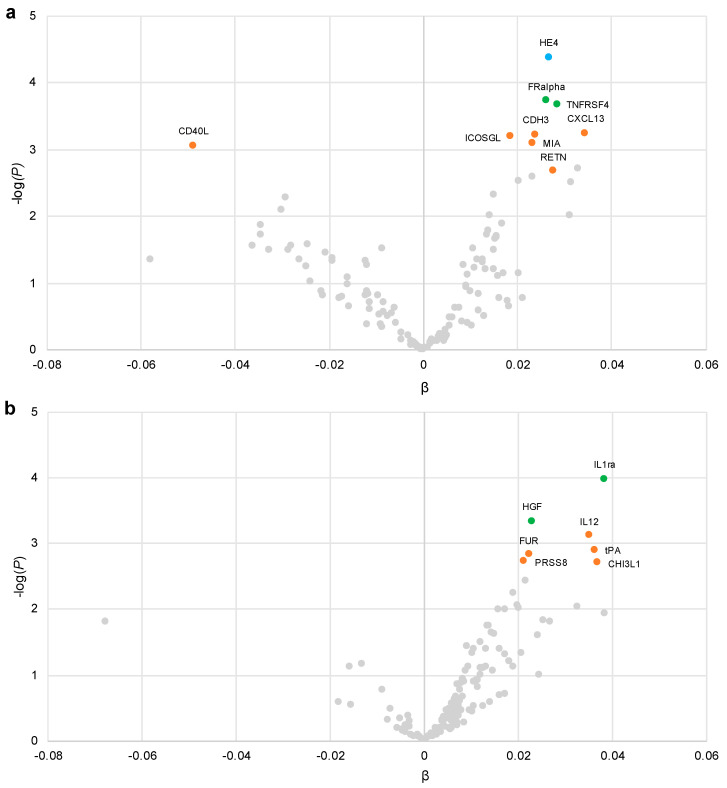
Volcano plot of associations between (**a**) added sugar intake (standardized) and (**b**) SSB intake (log transformed and standardized) and 136 plasma proteins in full sample analysis (*n* = 3351–4382). Linear regressions were adjusted for age, sex, season, screening date, total energy intake, education, smoking, alcohol and LTPA. Blue, TSIR replicated at α_1_ < FDR0.05; green, TSIR replicated at α_1_ < 0.01; orange, TSIR replicated at α_1_ < 0.05; grey, not TSIR replicated. FDR, false discovery rate; LTPA, leisure time physical activity; SSB, sugar-sweetened beverage; TSIR, two-step iterative resampling.

As presented in Table 3 and Figure 1a, at α_1_ < 0.05, nine proteins were internally replicated to associate with added sugar intake: epididymial secretory protein E4 (HE4), folate receptor alpha (FRalpha), tumor necrosis factor receptor superfamily member 4 (TNFRSF4), inducible T cell costimulator ligand (ICOGSL), CD40 ligand (CD40L), cadherin 3 (CDH3), C-X-C motif chemokine 13 (CXCL13), melanoma-derived growth regulatory protein (MIA) and resistin (RETN). Of these, HE4, FRalpha and TNFRSF4 remained internally replicated at α_1_ < 0.01, and HE4 was the only protein internally replicated at α_1_ < FDR0.05. Out of these nine plasma proteins, all except one (CD40L) were positively associated with added sugar intake.

### 3.3. Plasma Proteins Associated with SSB Intake

As presented in Table 3 and Figure 1b, at α_1_ < 0.05, seven proteins were internally replicated to associate with SSB intake: interleukin-1 receptor antagonist (IL1ra), hepatocyte growth factor (HGF), interleukin 12 (IL12), tissue-type plasminogen activator (tPA), prostasin (PRSS8), furin (FUR) and chitinase-3-like protein 1 (CHI3L1). IL1ra and HGF remained internally replicated at α_1_ < 0.01 and none of the proteins were internally replicated at α_1_ < FDR0.05. No protein was internally replicated to associate with both added sugar and SSB intake.

All 16 internally replicated proteins correlated significantly and positively with each other, with correlation coefficients ranging from 0.05 to 0.72, *p* < 0.001. A correlation matrix heatmap of all internally replicated proteins can be found in Figure S1. Figure S2 displays the potential pathways between the 16 identified proteins using the STRING database. A known interaction exists between RETN and CHI3L1, and co-expression occurs between HE4 and PRSS8, IL1ra and RETN, as well as between CD40L, TNFRSF4, CXCL13 and ICOSLG.

### 3.4. Associtions of Plasma Proteins with T2D Incidence

All seven proteins that were internally replicated to associate with SSB intake except for IL12 were strongly associated with increased T2D incidence (*p*-values of 6.9 × 10^−8^ to 2.8 × 10^−46^) (Table 4). Among the nine proteins associated with added sugar intake, CD40L and CXCL13 associated significantly with increased T2D incidence (*p*-values of 0.00045 and 0.00023). Notably, the highest significance for the added sugar-associated proteins (based on the *p*-value) was observed for CD40L, which was identified to associate inversely with added sugar intake. The associations were in general attenuated after adding baseline BMI and fasting glucose as covariates in the regression models (Table 4), but additional covariate adjustment for CRP only had minor impact (data not shown).

In an additional exploratory analysis aimed at finding potential interaction effects between added sugar and SSB intake, respectively, and the 136 plasma proteins on T2D risk, no significant interaction were observed after Bonferroni correction for multiple testing (Table S2).

### 3.5. Associations of Added Sugar and SSB Intake with CRP and T2D Incidence

**Figure 2:**
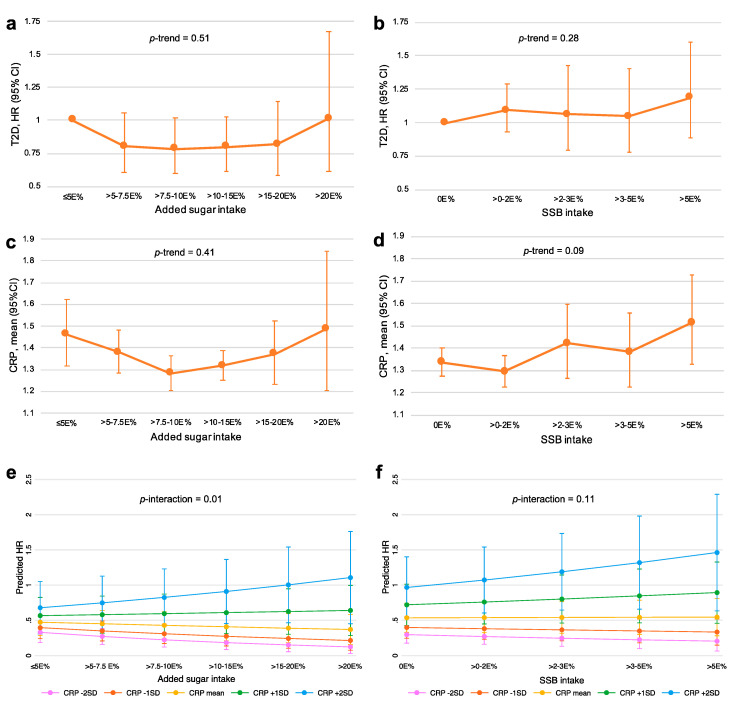
(**a**) Association between added sugar intake and T2D; (**b**) Association between SSB intake and T2D; (**c**) Association between added sugar intake and CRP; (**d**) Association between SSB intake and CRP; (**e**) Interaction between added sugar intake and CRP on T2D risk; (**f**) Interaction between SSB intake and CRP on T2D risk. Cox proportional hazards regressions and linear regressions were adjusted for age, sex, season, screening date, total energy intake, education, smoking, alcohol and LTPA. CRP was studied as log transformed and the predicted marginal means of CRP levels were exponentiated back for presentation. CRP, C-reactive protein; E%, percent of energy intake; HR, hazard ratio; LTPA, leisure time physical activity; SSB, sugar-sweetened beverage; T2D, type 2 diabetes.

As presented in Figure 2, no significant linear associations were observed between either added sugar intake or SSB intake and T2D risk (*p*-trend of 0.51 and 0.28, respectively) or CRP concentrations (*p*-trend of 0.41 and 0.09, respectively). Regarding added sugar intake, the lowest CRP concentrations and HRs for T2D incidence were observed in the middle intake categories, while for SSB intake, the associations appeared more linearly positive. In sensitivity analyses, excluding low and high energy reporters and past diet changers from these aforementioned analyses, the lowest T2D risk and CRP level were still observed in the middle categories of added sugar intake, suggesting U-shaped associations between added sugar intake and T2D and CRP (Table S3). Furthermore, a significant positive interaction between added sugar and CRP was observed on the association with T2D incidence (*p* = 0.01), where added sugar intake was positively associated with T2D at high CRP levels, but not associated at low CRP levels. A similar tendency was observed for SSB intake, but this interaction was not significant (*p* = 0.11).

## 4. Discussion

In the present study, we identified plasma proteins and proteomic signatures that cross-sectionally associated with added sugar and SSB intake. Most noteworthy is that we demonstrated that the SSB-associated proteins associated strongly with T2D incidence, indicating that SSB intake may be associated with a T2D-pathological proteomic signature, while such a signature was not observed for added sugar intake.

Using a TSIR approach, we identified nine plasma proteins that associated with added sugar intake after internal replication. Out of these, two proteins associated significantly with increased T2D incidence: CXCL13 and CD40L. We identified seven proteins that associated with SSB intake after internal replication and six of these were strongly associated with T2D incidence: HGF, tPA, CHI3L1, IL1ra, PRSS8 and FUR. These six associations were much stronger than those of the two proteins that associated with added sugar intake. This indicates that SSB intake associates with a T2D-pathological proteomic profile, while this cannot be stated for added sugar intake. The associations between the identified proteins and T2D were not independent of baseline BMI and fasting glucose levels (of varying degree), but all six SSB-associated proteins remained strongly associated with T2D incidence after adjustment.

In brief, among all of the 16 internally replicated proteins, CXCL13, IL12, IL1ra, ICOSLG, HGF, CD40L, CHI3L1 and RETN are involved, one way or another, in inflammatory response signaling. TNFRSF4 is involved in atherosclerosis development and tPA is involved in thrombolysis, while PRSS8, FRalpha, MIA, FUR, CDH3 and HE4 are involved in various cancer processes [34,35]. The protein FUR has previously been studied in depth in MDC-CC and found to be strongly associated with diabetes incidence [36], and HGF plays a role in both hepatic glucose and lipid metabolism, and in the development of insulin resistance, fatty liver disease, cardiovascular diseases and cancers [37,38]. Nevertheless, among these proteins, some could potentially promote inflammation or disease, while other could be involved in negative feedback signaling. Hence, we cannot say whether the observed associations are causal or if the proteins only serve as potential markers of high SSB intake and T2D risk.

The finding that none of the 16 internally replicated proteins overlapped as being associated with both added sugar intake and SSB intake further emphasizes the notion that added sugar intake and SSB intake are rather different exposures and cannot be equated [39,40,41]. Although, this discrepancy may be due to the fact that self-reporting of SSB intake potentially could be more accurate, because SSBs generally are consumed in standardized portion sizes, i.e., in cans and bottles, as compared to the total intake of added sugar. Further, on average, SSB intake only contributed to 10% of the energy to the total intake of added sugar. However, it is reassuring that proteins such as PRSS8, IL12, IL1ra, FUR and CXCL1 were associated with both added sugar and SSB intake, although not exceeding the cutoff for internal replication for both.

The previous epidemiological literature on diet and inflammatory markers has mostly focused on CRP. Intake of SSBs have frequently been associated with higher CRP [42,43,44,45,46,47]. However, to our knowledge, the only published epidemiological study that has studied the association between sugar intake and CRP concluded that such an association could only be found when separately studying sugar from liquid sources, not total intake of free sugars [48]. We could similarly not find any significant linear association between added sugar intake and CRP, but neither could we observe any significant association between SSB intake and CRP, although a tendency for such an association could be observed (*p*-trend = 0.09).

Intervention trials that have studied inflammatory markers after high sugar interventions have focused on comparing effects between fructose and glucose, and only rarely sucrose. All such trials were summarized in a systematic review where only CRP was found sufficiently studied to perform a meta-analysis, and no differential effects between different sugars could be seen [49]. In addition, what is noteworthy in the previous literature on diet and inflammation is that the highly used Dietary Inflammatory Index, which estimates the inflammatory potential of a diet, does not incorporate intake of either added sugar or SSBs in its computation [50].

No significant linear associations were observed for either added sugar intake or SSB intake on T2D incidence. However, SSB intake has previously been found to associate with T2D incidence in a sample of 25,069 participants in the full MDC cohort [51]. Hence, the reduced power of the current study (*n* = 4382) is a probable reason for the lack of an association. Since no significant associations were found, it was not meaningful to perform mediation analysis of the internally replicated plasma proteins. Nevertheless, a significant positive interaction effect between added sugar intake and CRP on the risk of T2D was observed (*p* = 0.01), where increased T2D risk from high added sugar intake could only be observed at higher CRP levels. Baseline CRP has previously been shown to associate strongly with the incidence of diabetes mellitus in the MDC-CC [52].

Tendencies for a U-shaped association between added sugar intake and incident T2D were observed, while more linearly positive tendencies were observed with SSB intake. A U-shaped association was also observed between added sugar and CRP, while the association between SSB and CRP appeared more direct. Furthermore, the relatively large number of plasma proteins that associated inversely with added sugar intake but not with SSB intake, as displayed in Figure 1, indicates a potentially less healthy profile among those consuming the least amount of added sugar and could have contributed to the increased T2D risk in this group. In previous analyses of the full MDC cohort, the highest HR for T2D were observed in the lowest quintile of added sugar intake [53]. Furthermore, U-shaped associations have been observed between added sugar intake (studied in the same six categories as in the present study) and mortality in the full MDC cohort [30]. The reason for this U-shape is thought to at least partially be due to potential dietary misreporting and reversed causation, where those at high risk, e.g., overweight individuals, have reduced their sugar consumption or at least reported their dietary intakes as if they had. This is probably a source of bias in our analyses, and it may also apply to our plasma protein identification approach, which assumes linearity. However, the U-shaped nature of the associations between added sugar and T2D and CRP remained after exclusion of low and high energy reporters and past diet changers. Additionally, the dietary assessment method has been validated and shown relatively high Pearson’s correlation coefficients regarding sugar intake (0.74 for women and 0.60 for men) [54].

A strength of this study is the use of the TSIR approach to identify plasma proteins. However, the main limitation is the lack of replication in external cohorts, which makes it difficult to make strong conclusions of any specific protein. Furthermore, the TSIR approach that was applied for internal replication has limitations, and it was modified to fit a more small-scale protein identification of 136 proteins rather than gene identification of millions of genes by lowering the α_1_ criteria. In addition, our study may suffer from insufficient statistical power in some of its analyses, especially for analyses of interaction and in small intake categories of added sugar and SSBs. Furthermore, the skewness of the SSB variable certainly complicates the performed analyses. Due to the large proportion of zero consumers, log transformation (with a small added constant) marginally improved the distribution. Further, since this is a middle-aged population studied in the 1990′s (born 1923–1950), the consumption habits may not be perfectly representative of a population of today. The large proportion of zero consumers of SSBs that were observed could be due to both underreporting and that consumption of SSBs might actually not have been very common in this population. This rather low overall SSB consumption (mean 1.2E%, median 0.07E%), as compared to what usually is observed in mainly American cohorts (and due to the skewness of the SSB variable), could have contributed to why we could not replicate an association between SSB intake and T2D, which so commonly is observed [9]. Finally, despite rigorous covariate adjustment, this is an epidemiological study where residual confounding cannot be ruled out and causality cannot be evaluated.

In conclusion, this is a hypothesis generating study that is the first of its kind to identify plasma proteins and proteomic signatures that are associated with added sugar and SSB intake. Turning to the proteome to elucidate the complex potential relationship between sugar intake and T2D incidence did not provide a clear enlightenment, but despite the fact that no significant associations were observed between added sugar and SSB intake with levels of CRP, the data suggests that SSB intake is related to a T2D-pathological proteomic signature, while this was not observed for added sugar intake. Replications in larger and more recent cohorts are necessary to verify our results.

## Figures and Tables

**Table 1:** Baseline characteristics of study participants across six categories of added sugar intake.

Added Sugar	≤5E%	>5–7.5E%	>7.5–10E%	>10–15E%	>15–20E%	>20E%
*n* = 4382	392	842	1129	1544	382	93
Sex, % women	60.5	62.7	61.0	62.1	61.0	54.8
University degree, %	15.1	14.6	13.4	10.2	5.8	6.5
Current smoker, %	32.7	26.1	24.9	24.6	28.3	41.9
Q5 alcohol, %	24.0	20.2	16.9	12.8	12.0	12.9
Incidence T2D, %	20.4	16.3	16.5	16.9	17.3	21.5
Age, years	56.0 (5.8)	56.5 (5.8)	57.7 (6.0)	57.7 (6.0)	58.1 (5.8)	57.7 (5.9)
BMI, kg/m^2^	26.2 (4.4)	25.7 (3.9)	25.4 (3.6)	25.4 (3.8)	25.0 (3.8)	25.2 (4.2)
Fasting glucose, mmol/L	5.66 (0.69)	5.65 (0.81)	5.58 (0.65)	5.60 (0.74)	5.60 (0.77)	5.65 (0.72)
CRP ^1,2^, nmol/L	1.5 (0.7–2.9)	1.3 (0.7–2.8)	1.2 (0.6–2.6)	1.2 (0.6–2.5)	1.4 (0.6–2.9)	1.65 (0.7–3.2)
Energy intake, kcal	2095 (718)	2225 (643)	2298 (636)	2381 (660)	2507 (708)	2572 (784)
SSB intake ^1^, E%	0 (0–0)	0 (0–0.5)	0 (0–1.1)	0.65 (0–2.3)	2.2 (0.4–4.9)	5.5 (1.4–11.1)
SSB:added sugar ^1^, E%	0 (0–0)	0 (0–8.3)	0 (0–12.3)	5.5 (0–18.4)	12.9 (2.4–29.8)	24.0 (6.2–45.0)

Data are expressed as percentages (categorical variables) or mean (SD) (continuous variables) unless stated otherwise. ^1^ Expressed as median (IQR). ^2^ *n* = 4291. BMI, body mass index; CRP, C-reactive protein; E%, percent of energy intake; IQR, interquartile range; SSB, sugar-sweetened beverage; T2D, type 2 diabetes; Q5, quintile 5 of consumers.

**Table 2:** Baseline characteristics of study participants across five categories of SSB intake.

SSBs	0E%	>0–2E%	>2–3E%	>3–5E%	>5E%
*n* = 4382	2039	1471	310	307	255
Sex, % women	62.7	61.5	64.2	57.0	54.5
University degree, %	13.5	11.7	8.4	8.5	7.5
Current smoker, %	27.1	24.3	30.0	25.7	28.2
Q5 alcohol, %	16.8	17.0	16.5	14.0	9.4
Incidence T2D, %	16.2	17.9	17.1	17.3	20.0
Age, years	57.6 (5.9)	57.0 (6.1)	56.9 (6.0)	57.2 (5.9)	57.4 (5.9)
BMI, kg/m^2^	25.4 (3.8)	25.3 (3.7)	25.6 (3.9)	25.8 (3.8)	26.2 (4.3)
Fasting glucose, mmol/L	5.60 (0.68)	5.59 (0.72)	5.63 (0.74)	5.64 (0.87)	5.75 (0.93)
CRP ^1,2^, nmol/L	1.3 (0.7–2.7)	1.2 (0.6–2.5)	1.35 (0.7–2.9)	1.4 (0.7–2.6)	1.5 (0.7–3.0)
Energy intake, kcal	2211 (649)	2422 (677)	2341 (636)	2456 (696)	2391 (711)
Added sugar intake, E%	8.6 (3.6)	9.99 (3.49)	11.5 (3.5)	13.0 (3.5)	16.4 (4.4)
SSB:added sugar ^1^, E%	0 (0–0)	8.22 (4.3–13.1)	22.2 (17.7–27.8)	29.6 (24.5–36.2)	47.3 (37.8–58.4)

Data are expressed as percentages (categorical variables) or mean (SD) (continuous variables) unless stated otherwise. ^1^ Expressed as median (IQR). ^2^ *n* = 4291. BMI, body mass index; CRP, C-reactive protein; E%, percent of energy intake; IQR, interquartile range; SSB, sugar-sweetened beverage; T2D, type 2 diabetes; Q5, quintile 5 of consumers.

**Table 3:** The number of times proteins associated with added sugar intake and SSB intake, respectively, out of 100 iterations of random discovery and replication cohorts at various α_1_ levels using a TSIR approach. A protein must be replicated at least 20 times to pass internal replication.

Added Sugar, E%	Replicated at α_1_ < 0.05	Replicated at α_1_ < 0.01	Replicated at α_1_ < FDR0.05
Epididymial secretory protein E4 (HE4)	69	56	20
Folate receptor alpha (FRalpha)	60	37	5
Tumor necrosis factor receptor superfamily member 4 (TNFRSF4)	51	27	3
Cadherin 3 (CDH3)	46	17	1
Inducible T Cell Costimulator Ligand (ICOSLG)	46	14	1
C-X-C motif chemokine 13 (CXCL13)	41	14	0
Melanoma-derived growth regulatory protein (MIA)	40	11	0
CD40 ligand (CD40L)	37	4	0
Resistin (RETN)	23	0	0
Immunoglobulin-like transcript 3 (ILT3)	19	0	0
Interleukin 12 (IL12)	17	0	0
Prostasin (PRSS8)	16	0	0
Matrix metalloproteinase-10 (MMP10)	12	1	0
C-X-C motif chemokine 1 (CXCL1)	3	0	0
Transforming growth factor alpha (TGFalpha)	2	0	0
Interleukin-1 receptor antagonist (IL1ra)	2	0	0
Adrenomedullin (AM)	1	0	0
Renin (REN)	1	0	0
Agouti-related protein (AGRP)	1	0	0
Cathepsin L1 (CTSL1)	1	0	0
Furin (FUR)	1	0	0
**SSB, E%**			
Interleukin-1 receptor antagonist (IL1ra)	60	46	5
Hepatocyte growth factor (HGF)	44	25	0
Interleukin 12 (IL12)	46	12	0
Prostasin (PRSS8)	31	1	0
Tissue-type plasminogen activator (tPA)	21	5	0
Furin (FUR)	24	0	0
Chitinase-3-like protein 1 (CHI3L1)	22	0	0
Cathepsin D (CTSD)	8	0	0
Tartrate-resistant acid phosphatase type 5 (TRAP)	1	0	0
Parkinson disease protein 7 (PARK7)	1	0	0
Proteinase-activated receptor 1 (PAR1)	1	0	0
Prolactin (PRL)	1	0	0
Lectin-like oxidized LDL receptor 1 (LOX1)	1	0	0
Myoglobin (MB)	1	0	0
C-X-C motif chemokine 1 (CXCL1)	1	0	0

Linear regressions were adjusted for age, sex, season, screening date, total energy intake, education, smoking, alcohol and LTPA. With the TSIR approach, the cutoff was always set to α_2_ < 0.05 in the replication cohorts. SSB intake is log transformed. FDR, false discovery rate; LTPA, leisure time physical activity; SSB, sugar-sweetened beverage; TSIR, two-step iterative resampling.

**Table 4:** Associations with T2D incidence for proteins internally replicated to associate with added sugar intake and SSB intake.

Added Sugar	*n*	Lifestyle Adjustments		Lifestyle Adjustments + BMI		Lifestyle Adjustments + BMI + Fasting Glucose	
		HR (95% CI)	*p*	HR (95% CI)	*p*	HR (95% CI)	*p*
HE4	4253	1.01 (0.93–1.10)	0.77	1.06 (0.97–1.16)	0.16	1.02 (0.93–1.11)	0.69
FRalpha	4253	0.94 (0.88–1.02)	0.15	1.00 (0.93–1.08)	0.98	0.92 (0.85–0.99)	0.029
TNFRSF4	4175	1.01 (0.93–1.09)	0.84	0.97 (0.90–1.05)	0.52	0.87 (0.81–0.95)	0.0012 *
CDH3	4241	0.96 (0.89–1.03)	0.23	0.98 (0.91–1.05)	0.52	0.94 (0.87–1.01)	0.11
ICOSLG	4253	1.04 (0.96–1.12)	0.35	1.06 (0.98–1.14)	0.15	0.95 (0.88–1.03)	0.25
CXCL13	4175	1.14 (1.06–1.22)	0.00045 *	1.12 (1.04–1.21)	0.0033	1.07 (0.98–1.16)	0.12
MIA	4252	0.96 (0.89–1.04)	0.34	1.01 (0.94–1.09)	0.79	0.99 (0.91–1.07)	0.74
CD40L	4382	1.15 (1.07–1.24)	0.00023 *	1.13 (1.05–1.22)	0.0012 *	1.08 (1.00–1.16)	0.047
RETN	4382	1.11 (1.03–1.19)	0.0057	1.08 (1.01–1.17)	0.031	1.12 (1.04–1.21)	0.0021 *
**SSBs**							
IL1ra	3761	1.51 (1.42–1.61)	4.6 × 10^−37^ *	1.35 (1.26–1.45)	2.1 × 10^−16^ *	1.27 (1.18–1.37)	6.6 × 10^−10^ *
HGF	4382	1.65 (1.53–1.77)	2.6 × 10^−38^ *	1.48 (1.37–1.60)	1.0 × 10^−22^ *	1.37 (1.27–1.48)	5.2 × 10^−15^ *
IL12	4252	1.05 (0.97–1.13)	0.24	0.98 (0.90–1.06)	0.55	0.87 (0.81–0.95)	0.0014 *
PRSS8	4252	1.43 (1.31–1.55)	7.7 × 10^−17^ *	1.34 (1.23–1.46)	8.2× 10^−12^ *	1.14 (1.05–1.24)	0.0030 *
tPA	4382	1.44 (1.34–1.55)	8.1 × 10^−22^ *	1.33 (1.23–1.43)	5.6 × 10^−13^ *	1.18 (1.09–1.28)	4.6 × 10^−5^ *
FUR	4253	1.78 (1.64–1.92)	2.8 × 10^−46^ *	1.54 (1.42–1.68)	4.9 × 10^−24^ *	1.30 (1.20–1.42)	2.2 × 10^−9^ *
CHI3L1	4370	1.22 (1.13–1.31)	6.9 × 10^−8^ *	1.17 (1.09–1.26)	2.1 × 10^−5^ *	1.17 (1.09–1.26)	1.5 × 10^−5^ *

Plasma proteins are standardized. Cox proportional hazards regressions were adjusted for age, sex, education, smoking, alcohol and LTPA (and BMI and fasting glucose in the additional models). * Significant after Bonferroni correction, *p* = 0.05/16 = 0.003. BMI, body mass index; HR, hazard ratio; LTPA, leisure time physical activity; SSB, sugar-sweetened beverage; T2D, type 2 diabetes.

## Supplementary Materials

The following are available online at https://www.mdpi.com/2072-6643/12/10/3129/s1, Figure S1: Correlation matrix of internally replicated proteins that associates with added sugar intake and SSB intake, Figure S2: Pathways between internally replicated proteins according to the STRING database 11.0, Table S1: List of all 136 studied plasma proteins, Table S2: Proteins with a nominally significant (*p* < 0.05) interaction with added sugar or SSB intake on T2D risk from an exploratory analysis of 136 plasma proteins, Table S3: Sensitivity analyses excluding low and high energy reporters and diet changers in analyses of associations between added sugar and SSB intake and CRP and T2D risk.

## References

[1] Minihane A.-M., Vinoy S., Russell W.R., Baka A., Roche H.M., Tuohy K.M., Teeling J.L., Blaak E.E., Fenech M., Vauzour D. (2015). Low-grade inflammation, diet composition and health: Current research evidence and its translation. Br. J. Nutr..

[2] Lyons C.L., Kennedy E.B., Roche H.M. (2016). Metabolic Inflammation-Differential Modulation by Dietary Constituents. Nutrients.

[3] Herter-Aeberli I., Gerber P., Hochuli M., Kohler S., Haile S.R., Gouni-Berthold I., Berthold H.K., Spinas G.A., Berneis K. (2011). Low to moderate sugar-sweetened beverage consumption impairs glucose and lipid metabolism and promotes inflammation in healthy young men: A randomized controlled trial. Am. J. Clin. Nutr..

[4] Sørensen L.B., Raben A., Stender S., Astrup A. (2005). Effect of sucrose on inflammatory markers in overweight humans. Am. J. Clin. Nutr..

[5] Kuzma J.N., Cromer G., Hagman D., Breymeyer K.L., Roth C.L., Foster-Schubert K.E., Holte S.E., Weigle D.S., Kratz M. (2016). No differential effect of beverages sweetened with fructose, high-fructose corn syrup, or glucose on systemic or adipose tissue inflammation in normal-weight to obese adults: A randomized controlled trial. Am. J. Clin. Nutr..

[6] Cox C.L., Stanhope K.L., Schwarz J.M., Graham J.L., Hatcher B., Griffen S.C., Bremer A.A., Berglund L., McGahan J.P., Keim N.L. (2011). Circulating Concentrations of Monocyte Chemoattractant Protein-1, Plasminogen Activator Inhibitor-1, and Soluble Leukocyte Adhesion Molecule-1 in Overweight/Obese Men and Women Consuming Fructose- or Glucose-Sweetened Beverages for 10 Weeks. J. Clin. Endocrinol. Metab..

[7] Silbernagel G., Machann J., Häring H.-U., Fritsche A., Péter A. (2013). Plasminogen activator inhibitor-1, monocyte chemoattractant protein-1, e-selectin and C-reactive protein levels in response to 4-week very-high-fructose or -glucose diets. Eur. J. Clin. Nutr..

[8] Buyken A.E., Goletzke J., Joslowski G., Felbick A., Cheng G., Herder C., Brand-Miller J.C. (2014). Association between carbohydrate quality and inflammatory markers: Systematic review of observational and interventional studies. Am. J. Clin. Nutr..

[9] Imamura F., O’Connor L., Ye Z., Mursu J., Hayashino Y., Bhupathiraju S.N., Forouhi N.G. (2015). Consumption of sugar sweetened beverages, artificially sweetened beverages, and fruit juice and incidence of type 2 diabetes: Systematic review, meta-analysis, and estimation of population attributable fraction. BMJ.

[10] Malik V.S., Popkin B.M., Bray G.A., Després J.-P., Willett W.C., Hu F.B. (2010). Sugar-Sweetened Beverages and Risk of Metabolic Syndrome and Type 2 Diabetes: A meta-analysis. Diabetes Care.

[11] Schwingshackl L., Hoffmann G., Lampousi A.-M., Knüppel S., Iqbal K., Schwedhelm C., Bechthold A., Schlesinger S., Boeing H. (2017). Food groups and risk of type 2 diabetes mellitus: A systematic review and meta-analysis of prospective studies. Eur. J. Epidemiol..

[12] Colditz G.A., Manson J.E., Stampfer M.J., Rosner B., Willett W.C., Speizer F.E. (1992). Diet and risk of clinical diabetes in women. Am. J. Clin. Nutr..

[13] Feskens E.J., Virtanen S.M., Räsänen L., Tuomilehto J., Stengård J., Pekkanen J., Nissinen A., Kromhout D. (1995). Dietary Factors Determining Diabetes and Impaired Glucose Tolerance: A 20-year follow-up of the Finnish and Dutch cohorts of the Seven Countries Study. Diabetes Care.

[14] Janket S.-J., Manson J.E., Sesso H., Buring J.E., Liu S. (2003). A prospective study of sugar intake and risk of type 2 diabetes in women. Diabetes Care.

[15] Barclay A.W., Flood V.M., Rochtchina E., Mitchell P., Brand-Miller J.C. (2007). Mappstat Glycemic Index, Dietary Fiber, and Risk of Type 2 Diabetes in a Cohort of Older Australians. Diabetes Care.

[16] Montonen J., Järvinen R., Knekt P., Heliövaara M., Reunanen A. (2007). Consumption of Sweetened Beverages and Intakes of Fructose and Glucose Predict Type 2 Diabetes Occurrence. J. Nutr..

[17] Boeing H., Schulz M., Heidemann C., Schienkiewitz A., Hoffmann K., Boeing H. (2008). Carbohydrate intake and incidence of type 2 diabetes in the European Prospective Investigation into Cancer and Nutrition (EPIC)-Potsdam Study. Br. J. Nutr..

[18] Sluijs I., Van Der Schouw Y.T., van der Daphne A.L., Spijkerman A.M., Hu F.B., Grobbee D.E., Beulens J.W. (2010). Carbohydrate quantity and quality and risk of type 2 diabetes in the European Prospective Investigation into Cancer and Nutrition–Netherlands (EPIC-NL) study. Am. J. Clin. Nutr..

[19] Heikkilä H.M., Schwab U., Krachler B., Männikkö R., Rauramaa R. (2012). Dietary associations with prediabetic states—The DR’s EXTRA Study (ISRCTN45977199). Eur. J. Clin. Nutr..

[20] Ahmadi-Abhari S., Luben R.N., Powell N., Bhaniani A., Chowdhury R., Wareham N.J., Forouhi N.G., Khaw K.-T. (2013). Dietary intake of carbohydrates and risk of type 2 diabetes: The European Prospective Investigation into Cancer-Norfolk study. Br. J. Nutr..

[21] Biggelaar L.J.C.J.D., Eussen S.J., Sep S.J.S., Mari A., Ferrannini E., Van Dongen M.C.J.M., Denissen K.F.M., Wijckmans N.E.G., Schram M.T., Van Der Kallen C.J. (2017). Associations of Dietary Glucose, Fructose, and Sucrose with β-Cell Function, Insulin Sensitivity, and Type 2 Diabetes in the Maastricht Study. Nutrients.

[22] Tasevska N., Pettinger M., Kipnis V., Midthune U., Tinker L.F., Potischman N., Neuhouser M.L., Beasley J.M., Van Horn L., Howard B.V. (2018). Associations of Biomarker-Calibrated Intake of Total Sugars with the Risk of Type 2 Diabetes and Cardiovascular Disease in the Women’s Health Initiative Observational Study. Am. J. Epidemiol..

[23] Meyer K.A., Kushi L.H., Jacobs D.R., Slavin J., Sellers T.A., Folsom A.R. (2000). Carbohydrates, dietary fiber, and incident type 2 diabetes in older women. Am. J. Clin. Nutr..

[24] Sartorelli D.S., Franco L., Gimeno S., Ferreira S.R.G., Cardoso M.A. (2009). Dietary fructose, fruits, fruit juices and glucose tolerance status in Japanese–Brazilians. Nutr. Metab. Cardiovasc. Dis..

[25] Hodge A.M., English D.R., O’Dea K., Giles G.G. (2004). Glycemic Index and Dietary Fiber and the Risk of Type 2 Diabetes. Diabetes Care.

[26] Hotamisligil G.S. (2006). Inflammation and metabolic disorders. Nat. Cell Biol..

[27] Donath M.Y., Shoelson S.E. (2011). Type 2 diabetes as an inflammatory disease. Nat. Rev. Immunol..

[28] Wang X., Bao W., Liu J., Ouyang Y.-Y., Wang D., Rong S., Xiao X., Shan Z.-L., Zhang Y., Yao P. (2012). Inflammatory Markers and Risk of Type 2 Diabetes: A systematic review and meta-analysis. Diabetes Care.

[29] Liu C., Feng X., Li Q., Wang Y., Li Q., Hua M. (2016). Adiponectin, TNF-α and inflammatory cytokines and risk of type 2 diabetes: A systematic review and meta-analysis. Cytokine.

[30] Ramne S., Dias J.A., González-Padilla E., Olsson K., Lindahl B., Engström G., Ericson U., Johansson I., Sonestedt E. (2018). Association between added sugar intake and mortality is nonlinear and dependent on sugar source in 2 Swedish population–based prospective cohorts. Am. J. Clin. Nutr..

[31] Mattisson I., Wirfält E., Aronsson C.A., Wallström P., Sonestedt E., Gullberg B., Berglund G. (2005). Misreporting of energy: Prevalence, characteristics of misreporters and influence on observed risk estimates in the Malmö Diet and Cancer cohort. Br. J. Nutr..

[32] Enhörning S., Sjögren M., Hedblad B., Nilsson P.M., Struck J., Melander O. (2016). Genetic vasopressin 1b receptor variance in overweight and diabetes mellitus. Eur. J. Endocrinol..

[33] Kang G., Liu W., Cheng C., Wilson C.L., Neale G., Yang J.J., Ness K.K., Robison L.L., Hudson M.M., Srivastava D.K. (2015). Evaluation of a two-step iterative resampling procedure for internal validation of genome-wide association studies. J. Hum. Genet..

[34] Szklarczyk D., Gable A.L., Lyon D., Junge A., Wyder S., Huerta-Cepas J., Simonovic M., Doncheva N.T., Morris J.H., Bork P. (2018). STRING v11: Protein–protein association networks with increased coverage, supporting functional discovery in genome-wide experimental datasets. Nucleic Acids Res..

[35] Olink Proteomics Complete Human Protein Biomarkers List. https://www.olink.com/products/complete-protein-biomarkers-list/.

[36] Fernandez C., Rysä J., Almgren P., Nilsson J., Engström G., Orho-Melander M., Ruskoaho H., Melander O. (2018). Plasma levels of the proprotein convertase furin and incidence of diabetes and mortality. J. Intern. Med..

[37] Oliveira A.G., Araújo T.G., Carvalho B.D.M., Rocha G.Z., Dos Santos A., Saad M.J.A. (2018). The Role of Hepatocyte Growth Factor (HGF) in Insulin Resistance and Diabetes. Front. Endocrinol..

[38] Madonna R., Cevik C., Nasser M., De Caterina R. (2012). Hepatocyte growth factor: Molecular biomarker and player in cardioprotection and cardiovascular regeneration. Thromb. Haemost..

[39] Johnson R.K., Appel L.J., Brands M., Howard B.V., Lefevre M., Lustig R.H., Sacks F., Steffen L.M., Wylie-Rosett J. (2009). Dietary Sugars Intake and Cardiovascular Health. Circulation.

[40] Wang J., Light K., Henderson M., O’Loughlin J., Mathieu M.-E., Paradis G., Gray-Donald K. (2013). Consumption of Added Sugars from Liquid but Not Solid Sources Predicts Impaired Glucose Homeostasis and Insulin Resistance among Youth at Risk of Obesity. J. Nutr..

[41] Welsh J.A., Wang Y., Figueroa J., Brumme C. (2018). Sugar intake by type (added vs. naturally occurring) and physical form (liquid vs. solid) and its varying association with children’s body weight, NHANES 2009-2014. Pediatr. Obes..

[42] De Koning L., Malik V.S., Kellogg M.D., Rimm E.B., Willett W.C., Hu F.B. (2012). Sweetened Beverage Consumption, Incident Coronary Heart Disease, and Biomarkers of Risk in Men. Circulation.

[43] Kosova E.C., Auinger P., Bremer A.A. (2013). The Relationships between Sugar-Sweetened Beverage Intake and Cardiometabolic Markers in Young Children. J. Acad. Nutr. Diet..

[44] Hert K.A., Fisk P.S., Rhee Y.S., Brunt A.R. (2014). Decreased consumption of sugar-sweetened beverages improved selected biomarkers of chronic disease risk among US adults: 1999 to 2010. Nutr. Res..

[45] Tamez M., Monge A., Lopez-Ridaura R., Fagherazzi G., Rinaldi S., Ortiz-Panozo E., Yunes E., Romieu I., Lajous M. (2018). Soda Intake Is Directly Associated with Serum C-Reactive Protein Concentration in Mexican Women. J. Nutr..

[46] Yu Z., Ley S.H., Sun Q., Hu F.B., Malik V.S. (2018). Cross-sectional association between sugar-sweetened beverage intake and cardiometabolic biomarkers in US women. Br. J. Nutr..

[47] Lin W.-T., Kao Y.-H., Sothern M.S., Seal D.W., Lee C.-H., Lin H.-Y., Chen T., Tseng T.-S. (2020). The association between sugar-sweetened beverages intake, body mass index, and inflammation in US adults. Int. J. Public Health.

[48] O’Connor L., Imamura F., Brage S., Griffin S.J., Wareham N.J., Forouhi N.G. (2017). Intakes and sources of dietary sugars and their association with metabolic and inflammatory markers. Clin. Nutr..

[49] Della Corte K.W., Perrar I., Penczynski K.J., Schwingshackl L., Herder C., Buyken A.E. (2018). Effect of Dietary Sugar Intake on Biomarkers of Subclinical Inflammation: A Systematic Review and Meta-Analysis of Intervention Studies. Nutrients.

[50] Shivappa N., Steck S.E., Hurley T.G., Hussey J.R., Hebert J.R. (2013). Designing and developing a literature-derived, population-based dietary inflammatory index. Public Health Nutr..

[51] Ericson U., Hindy G., Drake I., Schulz C.-A., Brunkwall L., Hellstrand S., Almgren P., Orho-Melander M. (2018). Dietary and genetic risk scores and incidence of type 2 diabetes. Genes Nutr..

[52] Bao X., Borné Y., Johnson L.S., Muhammad I.F., Persson M., Niu K., Engström G. (2018). Comparing the inflammatory profiles for incidence of diabetes mellitus and cardiovascular diseases: A prospective study exploring the ’common soil’ hypothesis. Cardiovasc. Diabetol..

[53] Olsson K., Ramne S., Gonzàlez-Padilla E., Ericson U., Sonestedt E. Associations of carbohydrates and carbohydrate-rich foods with incidence of type 2 diabetes. Br. J. Nutr..

[54] Riboli E., Elmståhl S., Saracci R., Gullberg B., Lindgärde F. (1997). The Malmo Food Study: Validity of two dietary assessment methods for measuring nutrient intake. Int. J. Epidemiol..

