# Supplementary material for: Identification of Inflammatory and Disease-Associated Plasma Proteins that Associate with Intake of Added Sugar and Sugar-Sweetened Beverages and Their Role in Type 2 Diabetes Risk
Source: Nutrients. 2020 Oct 14;12(10):3129. doi: 10.3390/nu12103129 (PMC7602152; doi:10.3390/nu12103129)
Supplement: Supplementary file 1 [file nutrients-12-03129-s001.pdf]

Supplementary Material

Supplementary Table 1. List of all 136 studied plasma proteins.

Name	Abbreviation	Name	Abbreviation
Agouti-related protein	AGRP	Interleukin-6	IL6
Adrenomedullin	AM	Interleukin-6 receptor subunit alpha	IL6RA
Amphiregulin	AR	Interleukin-7	IL7
B-cell activating factor	BAFF	Interleukin-8	IL8
Ovarian cancer-related tumor marker CA 125	CA125	Immunoglobulin-like transcript 3	ILT3
Carbonic anhydrase IX	CAIX	Integrin alpha-1	ITGA1
Caspase-3	CASP3	Melusin	ITGB1BP2
Caspase-8	CASP8	Kallikrein-6	KLK6
C-C motif chemokine 19	CCL19	Latency-associated peptide transforming growth factor beta-1	LAPTGFbeta1
C-C motif chemokine 20	CCL20	Leptin	LEP
C-C motif chemokine 3	CCL3	Lectin-like oxidized LDL receptor 1	LOX1
C-C motif chemokine 4	CCL4	Tyrosine-protein kinase Lyn	LYN
Tumor necrosis factor receptor superfamily member 5	CD40	Membrane-bound aminopeptidase P	mAmP
CD40 ligand	CD40L	Myoglobin	MB
Early activation antigen CD69	CD69	Monocyte chemotactic protein 1	MCP1
Cadherin-3	CDH3	Melanoma-derived growth regulatory protein	MIA
Cyclin-dependent kinase inhibitor 1	CDKN1A	MHC class I polypeptide-related sequence A	MICA
Chitinase-3-like protein 1	CHI3L1	Midkine	MK
Macrophage colony-stimulating factor 1	CSF1	Matrix metalloproteinase-1	MMP1
Cystatin-B	CSTB	Matrix metalloproteinase-10	MMP10
Cathepsin D	CTSD	Matrix metalloproteinase-12	MMP12
Cathepsin L1	CTSL1	Matrix metalloproteinase-3	MMP3
Fractalkine	CX3CL1	Matrix metalloproteinase-7	MMP7
C-X-C motif chemokine 1	CXCL1	Myeloperoxidase	MPO
C-X-C motif chemokine 10	CXCL10	NF-kappa-B essential modulator	NEMO
C-X-C motif chemokine 11	CXCL11	N-terminal pro-B-type natriuretic peptide	NTproBNP
C-X-C motif chemokine 13	CXCL13	NT-3 growth factor receptor	NTRK3
C-X-C motif chemokine 16	CXCL16	Osteoprotegerin	OPG
C-X-C motif chemokine 5	CXCL5	Pappalysin-1	PAPPA
C-X-C motif chemokine 6	CXCL6	Proteinase-activated receptor 1	PAR1
C-X-C motif chemokine 9	CXCL9	Parkinson disease protein 7	PARK7
Dickkopf-related protein 1	Dkk1	Platelet-derived growth factor subunit B	PDGFsubunitB
Eosinophil cationic protein	ECP	Platelet endothelial cell adhesion molecule	PECAM1
Epidermal growth factor	EGF	Placenta growth factor	PlGF
Epidermal growth factor receptor	EGFR	Prolactin	PRL
Eukaryotic translation initiation factor 4B	EIF4B	Prostasin	PRSS8
Extracellular matrix metalloproteinase inducer	EMMPRIN	P-selectin glycoprotein ligand 1	PSGL1
Epithelial cell adhesion molecule	EpCAM	Pentraxin-related protein PTX3	PTX3

Receptor tyrosine-protein kinase erbB-2	ErbB2HER2	Receptor for advanced glycosylation end products	RAGE
Receptor tyrosine-protein kinase erbB-3	ErbB3HER3	Regenerating islet-derived protein 4	REG4
Receptor tyrosine-protein kinase erbB-4	ErbB4HER4	Renin	REN
Endothelial cell-specific molecule 1	ESM1	Resistin	RETN
Ezrin	EZR	Stem cell factor	SCF
Fatty acid-binding protein, adipocyte	FABP4	E-selectin	SELE
FAS-associated death domain protein	FADD	SIR2-like protein 2	SIRT2
Tumor necrosis factor receptor superfamily member 6	FAS	Spondin-1	SPON1
Fas antigen ligand	FasL	Proto-oncogene tyrosine-protein kinase Src	SRC
Fibroblast growth factor 23	FGF23	ST2 protein	ST2
Fms-related tyrosine kinase 3 ligand	Flt3L	Tissue factor	TF
Folate receptor alpha	FRalpha	Transforming growth factor alpha	TGFalpha
Follistatin	FS	Thrombopoietin	THPO
Furin	FUR	Angiopoietin-1 receptor	TIE2
Galanin peptides	GAL	TIM-1	TIM
Galectin-3	Gal3	Thrombomodulin	TM
Growth/differentiation factor 15	GDF15	Tumor necrosis factor receptor 1	TNFR1
Growth hormone	GH	Tumor necrosis factor receptor 2	TNFR2
Heparin-binding EGF-like growth factor	HBEGF	Tumor necrosis factor receptor superfamily member 4	TNFRSF4
Epididymial secretory protein E4	HE4	Tumor necrosis factor ligand superfamily member 14	TNFSF14
Hepatocyte growth factor	HGF	Tissue-type plasminogen activator	tPA
Kallikrein-11	hK11	TNF-related apoptosis-inducing ligand	TRAIL
Heat shock 27 kDa protein	HSP27	TNF-related apoptosis-inducing ligand receptor 2	TRAILR2
Inducible T cell costimulator ligand	ICOSLG	TNF-related activation-induced cytokine	TRANCE
Interleukin-12	IL12	Tartrate-resistant acid phosphatase type 5	TRAP
Interleukin-16	IL16	Urokinase plasminogen activator surface receptor	UPAR
Interleukin-17 receptor B	IL17RB	Vascular endothelial growth factor A	VEGFA
Interleukin 18	IL18	Vascular endothelial growth factor D	VEGFD
Interleukin-1 receptor antagonist protein	IL1ra	Vascular endothelial growth factor receptor 2	VEGFR2
Interleukin-27 subunit alpha	IL27A	Vimentin	VIM

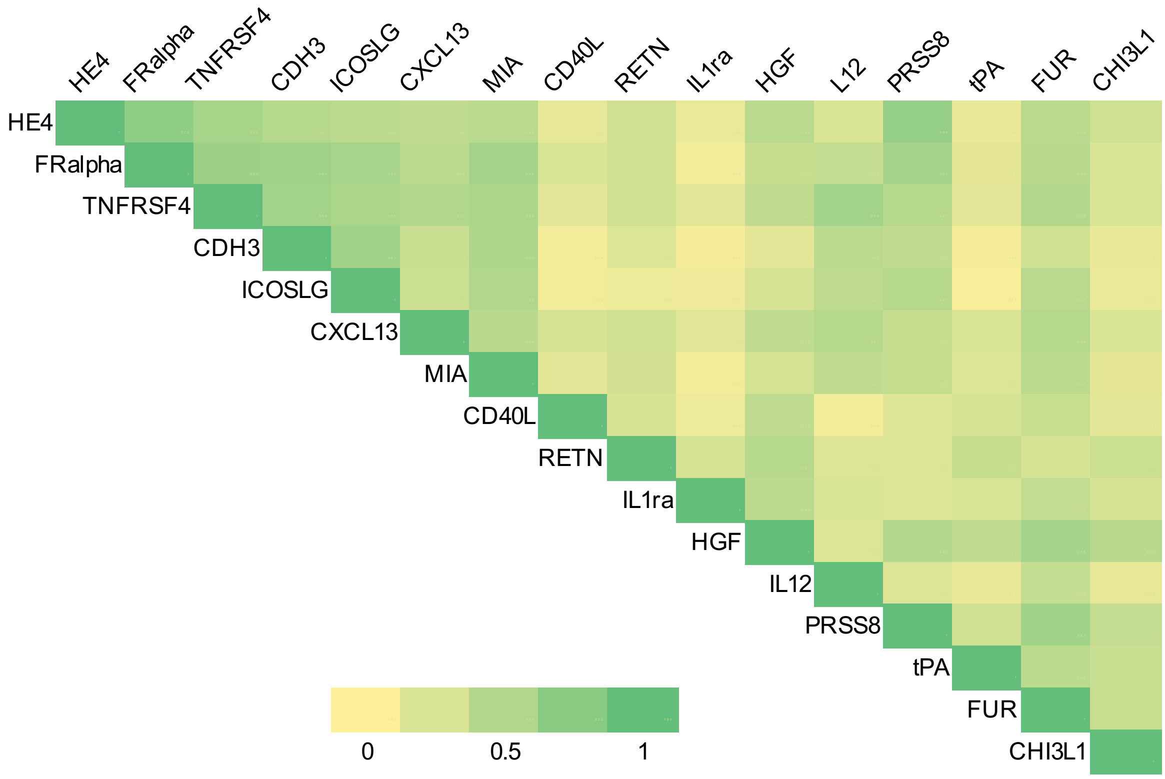

Supplementary Figure 1. Correlation matrix of internally replicated proteins that associates with added sugar intake and SSB intake. All correlations are positive and statistically significant *p*<0.001.

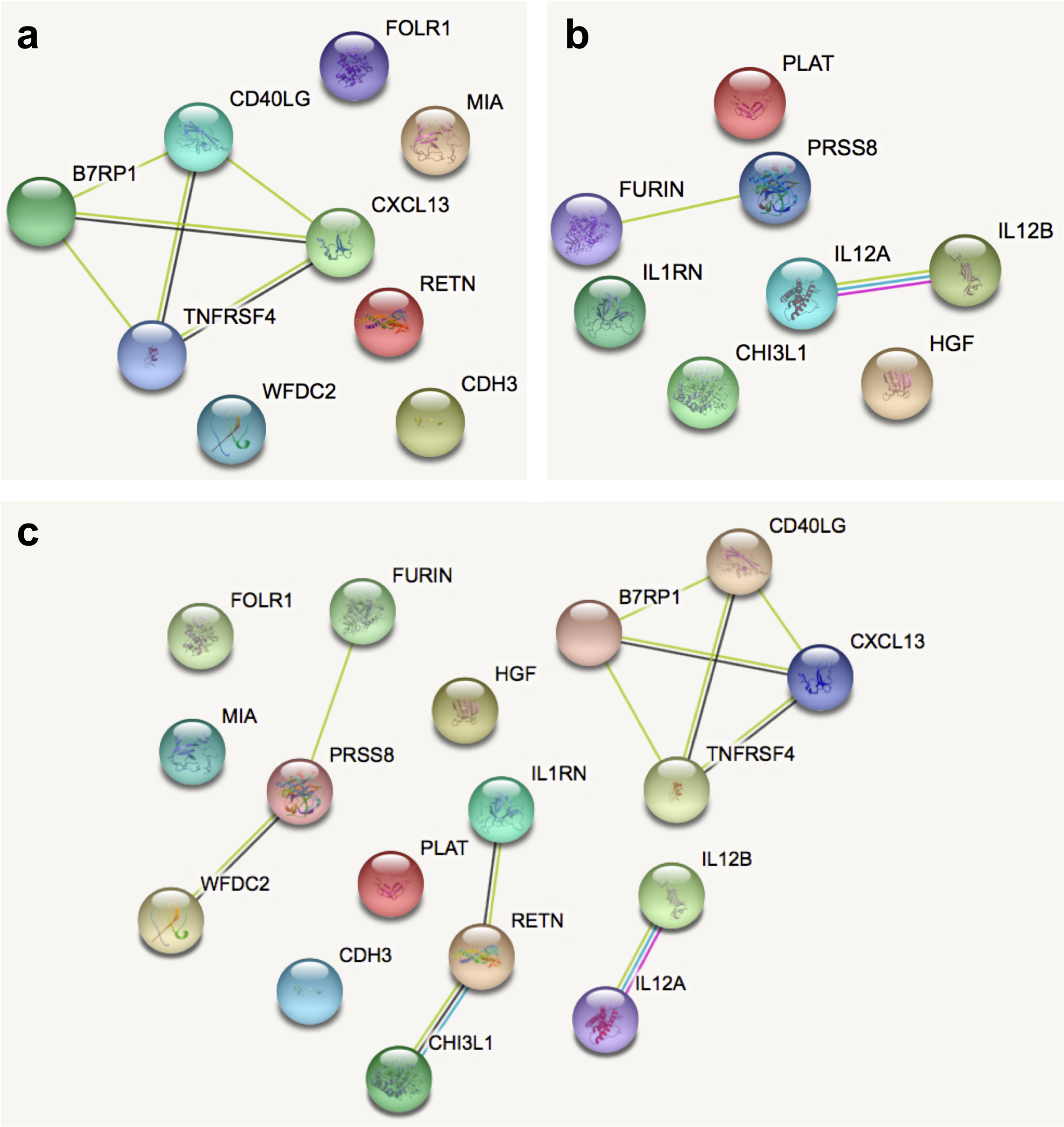

Supplementary Figure 2. Pathways between internally replicated proteins according to the STRING database 11.0. Blue lines represent known interactions from curated databases. (**a**) Proteins identified to associate with added sugar intake, (**b**) proteins identified to associate with SSB intake and (**c**) all 16 identified proteins that associate with added sugar or SSB intake. Pink lines represent known interactions determined experimentally. Black lines represent connection by co-expression. Green lines represent connection by textmining. Protein names and abbreviations from the STRING database is used here instead of the names used by Olink Proteomics: B7RP1=ICOSGL, CD40LG=CD40L, FOLR1=FRalpha, IL12A and IL12B=IL12, IL1Rn=IL1ra, PLAT=tPA, WFDC2=HE4.

Supplementary Table 2. Proteins with a nominally significant ($p<0.05$) interaction with added sugar or SSB intake on T2D risk from an exploratory analysis of 136 plasma proteins.

Lifestyle adjustments			Lifestyle adjustments + BMI		
**Added sugar**	**HR-interaction**	***p*-interaction**	**Added sugar**	**HR-interaction**	***p*-interaction**
MMP12	0.98	0.015	MMP10	0.98	0.007
REG4	0.98	0.022	AR	0.98	0.013
MMP10	0.98	0.028	hk11	0.98	0.014
CCL20	0.98	0.029	MIA	0.98	0.028
FRalpha	0.98	0.033	MMP12	0.98	0.029
AR	0.98	0.035	REG4	0.98	0.030
			TIM	0.98	0.035
			TRAP	0.98	0.040
			IL17RB	0.98	0.043
**SSB**			**SSB**		
MIA	0.92	0.0004	MIA	0.92	0.001
SCF	0.93	0.001	SCF	0.94	0.002
EMMPRIN	0.94	0.008	ICOSLG	0.94	0.009
PAPPA	1.06	0.011	Flt3L	0.94	0.015
IL18	1.06	0.014	PAPPA	1.06	0.016
ICOSLG	0.95	0.024	EMMPRIN	0.94	0.018
ST2	1.05	0.026	IL17RB	0.94	0.023
CXCL16	0.95	0.030	MMP10	0.95	0.023
Flt3L	0.95	0.031	ErbB3HER3	0.95	0.028
FRalpha	0.95	0.033	CD40L	1.05	0.034
Gal3	0.95	0.034	PDGFsubunitB	1.05	0.034
ErbB3HER3	0.95	0.040	ST2	1.05	0.041
PDGFsubunitB	1.05	0.041	CXCL16	0.95	0.043
MMP10	0.95	0.049			

Plasma proteins are standardized. Cox proportional hazards regressions were adjusted for age, sex, education, smoking, alcohol and LTPA (and BMI in the additional model). No interaction remained significant after Bonferroni correction for multiple testing at p-value=0.05/136=0.00037. BMI, body mass index; HR, hazard ratio; LTPA, leisure-time physical activity; SSB, sugar-sweetened beverage; T2D, type 2 diabetes.

Supplementary Table 3. Sensitivity analyses excluding low and high energy reporters and diet changers in analyses of associations between added sugar and SSB intake and CRP and T2D risk.

**T2D**		**HR (95% CI)**	**HR (95% CI)**	**HR (95% CI)**	**HR (95% CI)**	**HR (95% CI)**	**HR (95% CI)**	**HR-trend**	***p*-trend**
**Added sugar**	**n**	**≤5E%**	**>5-7.5E%**	**>7.5-10E%**	**>10-15E%**	**>15-20E%**	**>20E%**		
Full sample	4382	1	0.80 (0.61-1.06)	0.78 (0.60-1.02)	0.80 (0.62-1.03)	0.82 (0.58-1.14)	1.01 (0.62-1.67)	0.98 (0.92-1.04)	0.51
Excl low and high energy reporters	3538	1	0.85 (0.60-1.18)	0.78 (0.56-1.08)	0.81 (0.59 1.10)	0.80 (0.54-1.18)	1.01 (0.58-1.77)	0.97 (0.91-1.05)	0.49
Excl diet changers	3348	1	0.75 (0.53-1.05)	0.79 (0.58-1.09)	0.81 (0.60-1.11)	0.82 (0.55-1.22)	1.22 (0.71-2.10)	1.01 (0.93-1.08)	0.86
**SSB**		**0E%**	**>0-2E%**	**>2-3E%**	**>3-5E%**	**>5E%**			
Full sample	4382	1	1.10 (0.93-1.29)	1.06 (0.79-1.42)	1.05 (0.78-1.40)	1.19 (0.88-1.60)		1.03 (0.97-1.10)	0.28
Excl low and high energy reporters	3538	1	1.06 (0.88-1.28)	1.08 (0.78-1.51)	1.07 (0.77-1.47)	1.08 (0.76-1.53)		1.02 (0.95-1.10)	0.54
Excl diet changers	3348	1	1.13 (0.93-1.37)	1.01 (0.72-1.42)	1.10 (0.79-1.54)	1.27 (0.90-1.79)		1.05 (0.97-1.12)	0.21
**CRP***		**Mean (95% CI)**	**Mean (95% CI)**	**Mean (95% CI)**	**Mean (95% CI)**	**Mean (95% CI)**	**Mean (95% CI)**	**ß**	***p*-trend**
**Added sugar**		**≤5E%**	**>5-7.5E%**	**>7.5-10E%**	**>10-15E%**	**>15-20E%**	**>20E%**		
Full sample	4291	1.46 (1.32-1.62)	1.38 (1.28-1.48)	1.28 (1.21-1.36)	1.32 (1.25-1.39)	1.37 (1.23-1.53)	1.49 (1.20-1.84)	-0.01	0.41
Excl low and high energy reporters	3468	1.50 (1.32-1.71)	1.36 (1.26-1.48)	1.28 (1.19-1.37)	1.30 (1.23-1.38)	1.33 (1.18-1.49)	1.45 (1.15-1.83)	-0.02	0.25
Excl diet changers	3287	1.38 (1.22-1.56)	1.40 (1.29-1.52)	1.28 (1.19-1.37)	1.31 (1.24-1.39)	1.38 (1.22-1.55)	1.43 (1.13-1.82)	-0.01	0.63
**SSB**		**0E%**	**>0-2E%**	**>2-3E%**	**>3-5E%**	**>5E%**			
Full sample	4291	1.34 (1.28-1.40)	1.30 (1.23-1.37)	1.42 (1.26-1.60)	1.38 (1.23-1.56)	1.51 (1.33-1.73)		0.02	0.09
Excl low and high energy reporters	3468	1.32 (1.25-1.39)	1.29 (1.21-1.37)	1.42 (1.25-1.62)	1.35 (1.19-1.53)	1.48 (1.28-1.71)		0.02	0.17
Excl diet changers	3287	1.34 (1.27-1.41)	1.29 (1.21-1.37)	1.43 (1.26-1.63)	1.34 (1.17-1.53)	1.44 (1.24-1.67)		0.01	0.40

Cox proportional hazards regressions and linear regressions are adjusted for age, sex, education, smoking, alcohol and LTPA. *CRP was studied as log transformed and the predicted marginal means of CRP levels were exponentiated back for presentation. CRP, C-reactive protein; HR, hazard ratio; LTPA, leisure-time physical activity; SSB, sugar-sweetened beverage; T2D, type 2 diabetes.
